# In Situ NMR to Monitor Bulk Photopolymerization Kinetics

**DOI:** 10.1021/acsmacrolett.5c00171

**Published:** 2025-06-04

**Authors:** Luis L. Jessen, Kameron R. Hansen, George B. Crull, Tanner L. Grover, C. Allan Guymon

**Affiliations:** † Department of Chemical and Biochemical Engineering, 4083 The University of Iowa, Iowa City, Iowa 52242, United States; ‡ Department of Chemistry, 4083 The University of Iowa, Iowa City, Iowa 52242, United States; § Department of Chemical Engineering, 6756 Brigham Young University, Provo, Utah 84602, United States

## Abstract

The ability to precisely measure photopolymerization kinetics is paramount to controlling curing characteristics and material properties in photocurable systems. Traditional methods used to measure kinetics, such as real-time Fourier-transform infrared spectroscopy (RT-IR), are often limited by broadening mechanisms intrinsic to the system and poor spectral resolution. In this work, we present an *in situ* NMR spectroscopy technique to monitor bulk photopolymerization reactions wherein the polymer system is separated from the locking solvent via a capillary insert and photoexcited using an LED-coupled fiber optic. This technique allows for the isolated observation of the bulk reaction system while also benefiting from the high spectral resolution and rich chemical information offered by NMR. Relative rate constants (*k*_*p*_′) and ultimate monomer conversion were determined for four systems: neat hexyl acrylate, isobornyl methacrylate, *N*,*N*-dimethylacrylamide, and hexyl acrylate with cross-linker. By observing kinetic data of simple photopolymer systems, this work demonstrates the utility of *in situ* NMR photopolymerization as a complementary technique to conventional RT-IR and other methods for the kinetic monitoring of bulk photopolymer materials.

## Graphical abstract

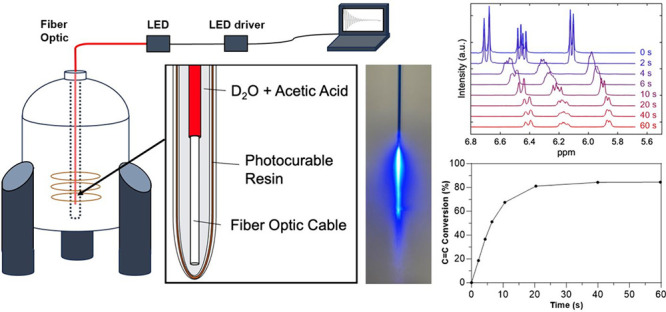

With its inherent high spatial and temporal control, photopolymerization has become a key curing technique for a variety of materials such as 3D-printed objects, photoresists, tissue scaffolds, biological coatings, and dental fillings, among many others.^1–5^ To control important material characteristics such as mechanical properties and morphology, a thorough understanding of polymerization kinetics is critical.^6–8^ Techniques to monitor these reaction kinetics can be divided into two categories, i.e., indirect and direct monitoring.

While useful, indirect techniques provide limited insight into the chemical makeup of a photopolymer system in real time. For example, photorheology is limited by measuring only the changes in mechanical properties during polymer formation while photodifferential scanning calorimetry simply determines the heat produced from the reaction exotherm.^9–11^ On the other hand, direct chemical measurements of molecular populations, such as that enabled by RT-IR, are capable of probing polymerization kinetics in real-time.^12–15^ Unfortunately, RT-IR often suffers from poor measurement resolution. Even relatively simple molecules have numerous IR-active vibrational modes which overlap due to thermal broadening at near-ambient temperatures.

*In situ* Nuclear Magnetic Resonance Spectroscopy (NMR) has been explored as a method for monitoring chemical reactions in real-time.^16–18^ Performing reactions directly inside the spectrometer eliminates the need for sample transfer and can provide information about short-lived transient reactive intermediate species while utilizing the high spectral resolution and rich chemical information that NMR provides.^19,20^ In recent years, several innovative designs have been used to guide light into the spectrometer to monitor light-induced chemical reactions.^21–24^ For example, Mills and co-workers inserted a fiber optic cable directly into an NMR sample tube containing both the locking solvent, CD_3_CN, and a photosensitive system consisting of oxygen-saturated toluene with a titania catalyst.^25^ Over a period of 3 h, concentrations of the different reagents, intermediates, products, and side products were monitored in real time, demonstrating the utility of *in situ* NMR in photochemistry. Additionally, *in situ* irradiation allowed time-domain measurements of spiropyran and its photoinduced metastable byproduct merocyanine, yielding kinetic quantities such as rate constants and half-lives.^26^

Beyond basic photochemical reactions, NMR has also been used to monitor photopolymerization reactions.^27–30^ For example, *in situ* NMR was used to study the photoinduced reaction of methyl acrylate in the presence of photocatalyst and a chain transfer agent by irradiating the top of a reaction solution inside the spectrometer using a Teflon NMR tube insert.^27^ By varying light intensities, linear rates of polymerization were observed as predicted by the steady-state assumption and used to determine polymerization rate constants. Temporal control of the reaction was demonstrated by turning the light source on and off in automated intervals. Liu and co-workers further demonstrated the utility of monitoring photopolymerizations *ex situ* (removing sample from the spectrometer for irradiation) using NMR by determining reactivity ratios of the copolymerization of methyl methacrylate and 2-(dimethylamino)­ethyl methacrylate dissolved in CDCl_3_.^29^ Conventionally, reactivity ratios are obtained by examining mathematical best fits based on feed ratios (*f*_1_) and polymer compositions (*F*_1_). However, both quantities typically require the interruption of the reaction to determine multiple compositional measurements and are restricted to low degrees of monomer conversion.^31–33^ The broad spectral resolution of NMR enabled the direct measurement of both *f*_1_ and *F*_1_ even at high degrees of monomer conversion, thus allowing for the direct determination of reactivity ratios. While these studies demonstrate the utility of NMR as a tool for kinetic measurements, they generally require the dissolution of the reaction system in deuterated solvent for spectrometer locking. As kinetic parameters are generally affected by solvent-monomer interactions, producing accurate predictions of kinetic behavior of bulk photopolymerization reactions is therefore challenging on the basis of data collected in solution.

In this work, we employed *in situ* NMR to monitor the kinetic evolution of the solvent-free bulk photopolymerization of linear and cross-linked hexyl acrylate (HA), *N*,*N*-dimethylacrylamide (AAm), and isobornyl methacrylate (IBOMA) systems using a fiber optic cable to direct UV-light into the spectrometer (Figure S1). Monomer conversion was monitored, and relative polymerization parameters were obtained from conversion data. Through examination of a broad range of bulk monomer photopolymerizations *in situ*, this NMR technique has provided a new perspective for observing photopolymer reactions and has demonstrated its potential as a powerful and complementary tool to examine photopolymerization kinetics.

To demonstrate the utility of NMR in monitoring polymerization kinetics of bulk polymer resins, we designed an *in situ* NMR apparatus wherein the resin is separated from the locking solvent using concentric inner capillaries (see Figure 1). A fiber optic cable directed UV-light into the inner capillary (O.D. = 4 mm) which was placed within the NMR outer tube (O.D. = 5 mm). The annulus between the tubes contained the bulk photopolymerizable resin at a thickness of approximately 100 μm, on the order of the thickness of typical photopolymer thin film systems.^34,35^ Systems were photoexcited by a frosted fiber optic tip to produce a uniform light intensity at the resin interface (see Methods, SI, Figure S2). In addition to housing the fiber optic, the concentric capillary also contained the locking solvent (D_2_O) and internal standard (acetic acid).

**Figure 1:**
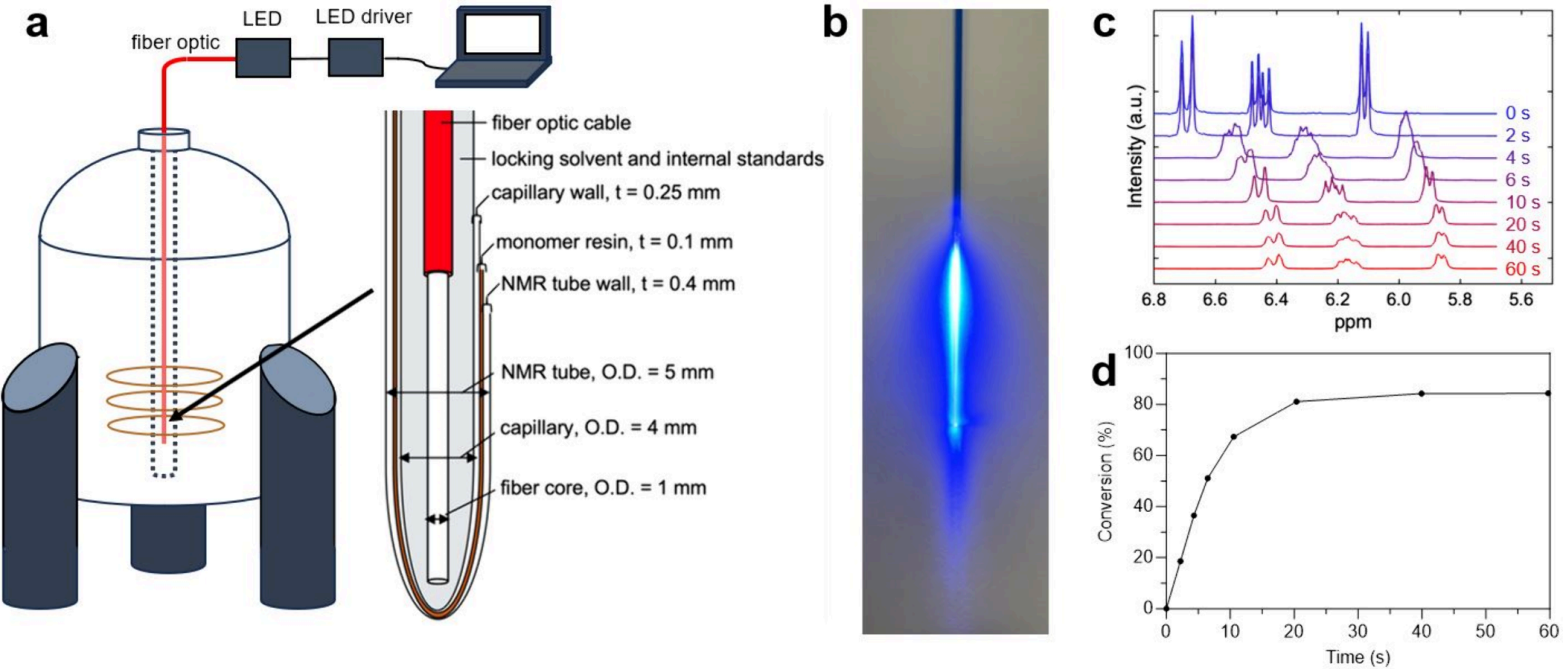
(a) Depiction of *in situ* NMR photocuring apparatus, where t represents the respective thickness of the various elements, and (b) photo of fiber optic cable emitting light inside concentric inner capillary. (c) Consecutive NMR spectra showing the disappearance and upfield shift of vinylic proton resonance from hexyl acrylate. (d) Conversion profile obtained from quantifying the area of the vinylic proton resonance of hexyl acrylate as a function of light exposure time.

Samples were exposed to light for defined intervals to initiate the polymerization. NMR spectra, collected between light illumination periods, were collected and analyzed to determine monomer conversion as a function of light exposure time. Figure 1c shows the disappearance of the vinylic proton resonances of hexyl acrylate monomer at ∼ 6 ppm in the spectrum throughout the course of the polymerization. As acrylate double bonds are consumed, both carbon atoms transition from sp^2^ to sp^3^ hybridization, forming the backbone of the polymer and driving a decrease in the vinylic resonance.^36^ This change reduces the NMR peak area by the amount proportional to the decrease in the acrylate double bond population, and hence, the unreacted hexyl acrylate monomer population. In addition to a reduction in area, slight peak broadening and upfield shift of the peaks were observed as a result of the decrease in monomer mobility due to the increase in polymer molecular weight and viscosity. Using the constant resonances of reference standards contained within the concentric inner capillary, the vinyl peak areas were then normalized and integrated to create a conversion profile (*X*) via eq S1. The conversion profile, shown in Figure 1d, follows the progression of the photopolymerization reaction. Remarkably, despite significantly increased viscosity expected at higher conversion, a high signal-to-noise (S/N) ratio was maintained throughout the reaction by optimizing instrument parameters to capture relaxations both in the initial resin as well as the final polymeric state (see Methods, SI). Low relative error between experiments (relative standard deviation approximately 1.3%) showed that high reproducibility can be achieved.

To evaluate if this same peak-identification and integration method for tracking the hexyl acrylate monomer concentration is possible using other nuclei, independent ^13^C-spectra were collected and analyzed in a similar fashion. The resonances corresponding to the two double bond carbons appear between 129 and 130 ppm in the ^13^C spectrum (Figure 2a).^37^ These peaks exhibited area decrease, broadening, and upfield shift concomitant with the light exposure time, similar to those previously analyzed in the ^1^H spectra (Figure 2b). Integration of the peak areas yielded a conversion profile that closely resembles that of the ^1^H-spectrum (Figure 2c). In repeating this experiment, the ^13^C-spectrum consistently showed a slightly higher conversion than the ^1^H-spectrum. This small increase is likely a result of the significantly longer experimental collection time required for ^13^C-spectra (13 min), in contrast to ^1^H-spectra (1.5 min). As a result, polymerization could potentially continue even without light exposure during collection of the ^13^C-spectra. This dark cure effect might be mitigated by including relaxation agents within the resin to reduce the required collection time of ^13^C-spectra and thus collect each data point more rapidly.

**Figure 2:**
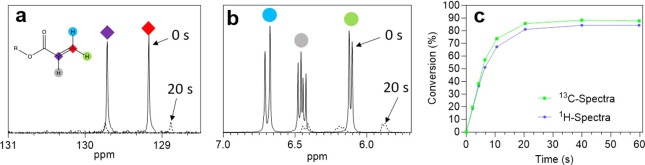
(a) ^1^H- and (b) ^13^C-spectra show the disappearance of vinylic resonances for both nuclei during the course of the bulk photopolymerization of hexyl acrylate. (c) Conversion profiles obtained from monitoring the vinylic resonances in ^1^H- and ^13^C-spectra using *in situ* NMR photopolymerization.

To investigate whether the polymerization kinetics, as measured by NMR, are consistent with typical photopolymerization dependence on light exposure, double bond conversion was also determined as a function of time and light intensity.^38,39^ Conversion profiles were measured using the ^1^H spectrum at four different light intensities (Figure S3). By assuming a steady state of the radical concentration during the initial stages of the reaction, these conversion profiles can be linearized using a logarithmic form of monomer conversion (Figure 3a). Further assuming that the propagation rate constant (*k*_*p*_), initiator efficiency (*f*), quantum yield (φ), light intensity (*I*_0_), and the termination rate constant (*k*_*t*_) remain largely unchanged during the initial stages of the reaction, these values can be combined to form an apparent rate constant *k*_*p*_′:^38^

1 $${k_{p}}^{\prime }=k_{p}{\left(\frac{f\varphi I_{a}}{k_{t}}\right)}^{1/2}$$

The values for *k*_*p*_′ of each light intensity can thus be found in the slope of the linearized conversion graph. As the light intensity is increased from 0.53 to 0.95 mW/cm^2^, we observe *k*_*p*_′ rise from approximately 0.041 to 0.067 s^–1^, almost a 70% increase. A further increase to 1.13 mW/cm^2^ results in another 30% gain in *k*_*p*_′. Finally, progressing to the highest light intensity of 1.72 mW/cm^2^, results in another 10% increase in *k*_*p*_′. As a result of bimolecular termination in free radical polymerizations, *k*_*p*_′ is expected to be linearly proportional to the square root of light intensity (eq 1).^38^ This relationship was clearly observed in Figure 3b where the values found for *k*_*p*_′ show a linear relationship to *I*_0_^1/2^.

**Figure 3:**
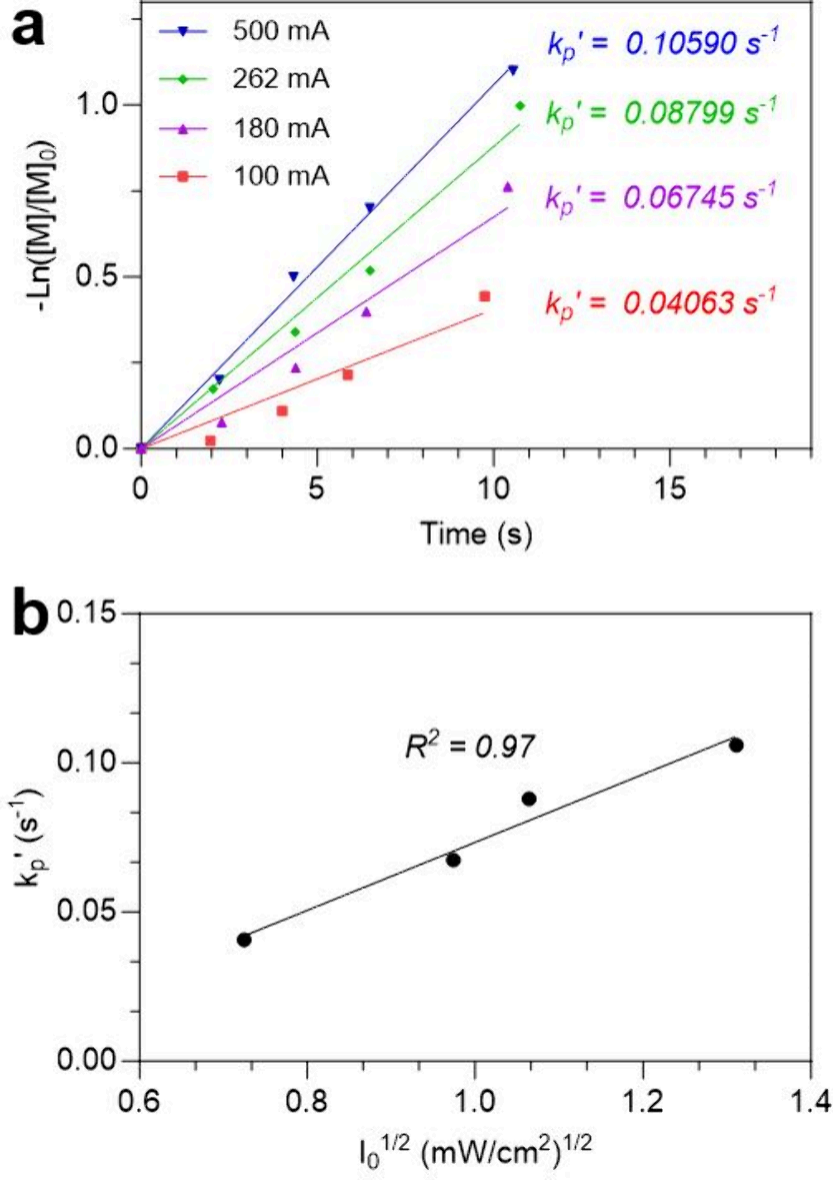
(a) Linearized conversion data of hexyl acrylate polymerizations at four different light intensities. By controlling incident light intensity, the rate of polymerization can be adjusted to reveal the apparent rate constant *k*_*p*_′. (b) As predicted theoretically, *k*_*p*_′ is linearly related to the square root of incident light intensity.

This ability to predict kinetic rate parameters even within a relatively narrow range of light intensities illustrates the utility of *in situ* NMR photopolymerization to monitor polymerization kinetic behavior.

Radical-based photopolymerizations frequently involve the use of a variety of different monomeric functional groups such as acrylates, methacrylates, and acrylamides, among others. To evaluate the ability to measure photopolymerization kinetics of monomers other than acrylates, we applied this *in situ* NMR technique to study N,N-dimethylacrylamide (AAm) and isobornyl methacrylate (IBOMA). Figure 4a and b show the curing-induced evolution of the spectral regions containing the vinylic protons (5 – 7.5 ppm) of AAm and IBOMA, respectively. In the resin state, the AAm resonances displayed a slight upfield shift compared to their HA counterparts due to the less electron-withdrawing nitrogen atom in place of an oxygen on the acrylic ester. IBOMA, on the other hand, showed only two singlets corresponding to the two protons on the sp^2^-hybridized beta-carbon. As the polymerization proceeded and monomer was consumed, the peaks associated with the double bond exhibited the expected area decrease, broadening, and upfield shift observed previously in the HA system. The conversion profiles from integrating the peak areas as a function of time are presented in Figure 4a,b. The conversion for the first 6 s is in good agreement with the steady-state approximation and biomolecular termination, as a linear fit between −ln­([M]/[M_0_]) and time resulted in *R* ^2^ values of 0.99 and 0.96 for IBOMA and AAm, respectively (Figure S4). After 10 s, however, in contrast to the HA system, both spectra showed a complete disappearance of all peaks except for those from components within the concentric inner capillary (acetic acid and H_2_O).

**Figure 4:**
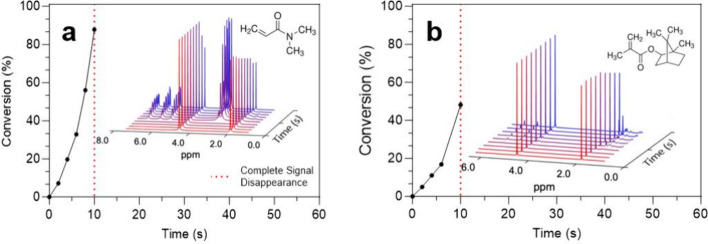
(a) Conversion profiles of *N*,*N*-dimethylacrylamide (AAm) and (b) isobornyl methacrylate (IBOMA) as a function of exposure time, as determined by *in situ* NMR photopolymerization.

At this stage, shimming the NMR magnet became challenging, often taking several minutes, and requiring manual phase corrections for each data point to enable data analysis. This change was likely a result of the restricted mobility of the polymer chains cured to high conversion; however, the same trend was not observed for HA. The most likely cause for this behavior was the lower inherent glass-transition temperature (*T*_g_) of pHA (−45 °C) compared to both pIBOMA (120 °C) and pAAm (52 °C), permitting more molecular motion and thus enabling residual unreacted double bonds to relax/emit signal even late into the reaction.^40–42^ Since both AAM and IBOMA moieties did not show any resonance aside from those inside the concentric capillary at the end of the reaction, their final conversion could thus not be determined.

To contrast such behavior with a lightly cross-linked system which should also have more limited mobility, a small amount of cross-linking PEGDA (2.5 wt %) was added to HA. This network forming system resulted in significantly higher ultimate conversion of (∼98%) than in neat HA. Despite higher conversion and more restricted mobility compared to linear HA, broad peaks corresponding to the polymer backbone were still observed even at the very end of the reaction (Figure 5). Additionally, shimming and phase corrections were relatively unaffected even during the late stages of the reaction. Thus, it appears that high *T*_g_s of the forming polymer can significantly limit the ability to collect spectra late into the reaction of bulk photopolymerizations, potentially requiring an increase in temperature for accurate conversion measurements of higher *T*_g_ systems. Additionally, magic-angle spinning solid-state NMR may be useful in examining kinetics at high conversion in high-*T*_g_ systems by allowing highly resolved spectra of brittle solids.^30^

**Figure 5:**
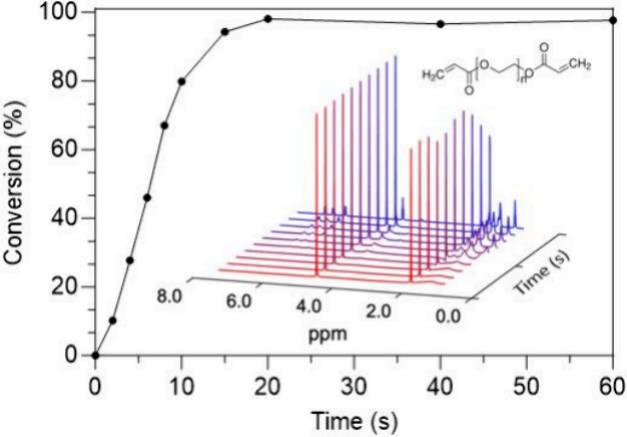
Monomer conversion profiles of 2.5 wt % polyethylene glycol diacrylate (PEGDA) in hexyl acrylate (HA) as a function of exposure time, as determined by *in situ* NMR photopolymerization.

In this work, bulk photopolymerization reactions were monitored by NMR spectroscopy (*in situ* NMR). Light was delivered via a fiber optic cable into a transparent concentric inner capillary immersed in photocurable resins consisting of acrylates, acrylamides, and methacrylates with the locking solvent contained within the inner capillary. ^1^H- and ^13^C-spectra were collected to monitor reaction kinetics by following the disappearance of vinylic resonances used to calculate monomer conversion as a function of time. Conversion profiles stemming from ^1^H- and ^13^C-spectra were in agreement resulting in nearly identical conversions at each time point. Early stages in the reaction during which steady state could be assumed were linearized to determine apparent rate constants, *k*_*p*_′. By adjusting incident light intensity over four levels, *k*_*p*_′ showed a linear relationship to *I*_0_^1/2^, as predicted theoretically. Varying monomer species showed a clear spectral acquisition dependence in later stages of the reaction on the glass-transition temperature (*T*_g_) of the forming polymer. While residual monomer and polymer resonances were visible even at high conversions in low-*T*_g_ systems, neither monomer nor polymer resonances were visible at this stage for high-*T*_g_ systems such as IBOMA and AAm. This study demonstrates the utility of *in situ* NMR photopolymerization to monitor bulk photopolymer reactions and thus expands the toolbox of available techniques to measure reaction kinetics of solvent-free photopolymer resin systems which could easily also be adapted to other bulk polymerization systems. Furthermore, the broad spectral field available for analysis as well as the ability to measure different nuclei in the same reaction potentially enable the *in situ* kinetic monitoring of complex processes such as copolymerizations, photopolymerization-induced phase separation (PhIPS), tacticity determination, and *in situ* photoinitiator consumption, to name a few.

## Supplementary Material



## Notes

Experimental details on materials, methods, and instrument parameters; Picture of in situ NMR apparatus; Graphic depicting light intensity measurements on surface of concentric inner capillary; Figure showing double bond conversion as a function of time at different light intensities; Figure showing linearized conversion profiles of *N*,*N*-dimethylacrylamide (AAm) and isobornyl methacrylate (IBOMA) monomer (PDF)

## References

[1] Topa-Skwarczyńska M., Jankowska M., Gruchała-Hałat A., Petko F., Galek M. (2023). High-performance photoinitiating systems for new generation
dental fillings. Dental Materials.

[2] Wake M. C., Patrick C. W., Mikos A. G. (1994). Original
Contribution Pore Morphology
Effects On The Fibrovascular Tissue Growth In Porous Polymer Substrates. Cell Transplant.

[3] Faxälv L., Ekblad T., Liedberg B., Lindahl T. L. (2010). Blood compatibility
of photografted hydrogel coatings. Acta Biomaterialia.

[4] Dong H., Kong Q., Jiang S., Cheng J., Zhang Q. (2024). Customized Preparation
of Heat-Resistant Fully Flexible Sensors Based
on Coaxial 3D Printing. ACS Appl. Mater. Interfaces.

[5] Green B. J., Guymon C. A. (2019). Modification of
mechanical properties and resolution
of printed stereolithographic objects through RAFT agent incorporation. Additive Manufacturing.

[6] Anastasio R., Peerbooms W., Cardinaels R., Van Breemen L. C. A. (2019). Characterization
of Ultraviolet-Cured Methacrylate Networks: From Photopolymerization
to Ultimate Mechanical Properties. Macromolecules.

[7] Howard B., Wilson N. D., Newman S. M., Pfeifer C. S., Stansbury J. W. (2010). Relationships
between conversion, temperature and optical properties during composite
photopolymerization. Acta Biomateralia.

[8] Aguiar T. R., De Oliveira M., Arrais C. A. G., Ambrosano G. M. B., Rueggeberg F. (2015). The effect of photopolymerization on the
degree of conversion, polymerization kinetic, biaxial flexure strength,
and modulus of self-adhesive resin cements. Journal of Prosthetic Dentistry.

[9] Bachmann J., Schmölzer S., Ruderer M. A., Fruhmann G., Hinrichsen O. (2022). Photo-differential
scanning calorimetry parameter study of photopolymers used in digital
light synthesis. SPE Polymers.

[10] Grover T. L., Guymon C. A. (2024). Effect of Block
Copolymer Self-Assembly on Phase Separation
in Photopolymerizable Epoxy Blends. Macromolecules.

[11] Corder R. D., Dudick S. C., Bara J. E., Khan S. A. (2020). Photorheology and
Gelation during Polymerization of Coordinated Ionic Liquids. ACS Applied Polymer Materials.

[12] Gorsche C., Harikrishna R., Baudis S., Knaack P., Husar B. (2017). Real Time-NIR/MIR-Photorheology:
A Versatile Tool for the in Situ
Characterization of Photopolymerization Reactions. Anal. Chem..

[13] Hasa E., Scholte J. P., Jessop J. L. P., Stansbury J. W., Guymon C. A. (2019). Kinetically Controlled Photoinduced Phase Separation
for Hybrid Radical/Cationic Systems. Macromolecules.

[14] Grover T. L., Guymon C. A. (2023). Controlling network
morphology in hybrid radical/cationic
photopolymerized systems. Polym. Chem..

[15] Jessen L. L., Grover T. L., Oberbroeckling N. V., Willemse R., Guymon C. A. (2025). Enhancing
the Thermomechanical Properties of Photopolymer Networks Using Small-Molecular-Weight
Hyperbranched Prepolymer Additives. Macromolecules.

[16] Torres A., Molina Perez N., Overend G., Hodge N., Heaton B. T. (2012). High-pressure
in situ NMR methods for the study of reaction kinetics
in homogeneous catalysis. ACS Catal..

[17] Cha M., Shin K., Lee H., Moudrakovski I. L., Ripmeester J. A. (2015). Kinetics of methane
hydrate replacement with
carbon dioxide and nitrogen gas mixture using in situ NMR spectroscopy. Environ. Sci. Technol..

[18] Stepanov A. G., Parmon V. N., Freude D. (2007). In situ NMR spectroscopy in heterogeneous
catalysis: Kinetic study of hydrocarbon conversion mechanisms. Kinetics and Catalysis.

[19] Closs G. L., Closs L. E. (1969). Induced Dynamic Nuclear Spin Polarization
in Photoreductions
of Benzophenone by Toluene and Ethylbenzene1. UTC. J. Am. Chem. Soc..

[20] Closs G. L., Closs L. E. (1969). Induced Dynamic Nuclear Spin Polarization in Photoreductions
of Benzophenone by Toluene and Ethylbenzene1. J. Am. Chem. Soc..

[21] Feldmeier C., Bartling H., Riedle E., Gschwind R. M. (2013). LED based NMR illumination
device for mechanistic studies on photochemical reactions - Versatile
and simple, yet surprisingly powerful. J. Magn.
Reson..

[22] Seegerer A., Nitschke P., Gschwind R. M. (2018). Combined In Situ Illumination-NMR-UV/Vis
Spectroscopy: A New Mechanistic Tool in Photochemistry. Angew. Chem., Int. Ed..

[23] Okuno Y., Cavagnero S. (2016). Fluorescein: A Photo-CIDNP Sensitizer Enabling Hypersensitive
NMR Data Collection in Liquids at Low Micromolar Concentration. J. Phys. Chem. B.

[24] Prochazkova E., Cechova L., Kind J., Janeba Z., Thiele C. M., Dracinsky M. (2018). Photoswitchable Intramolecular
Hydrogen Bonds
in 5-Phenylazopyrimidines Revealed By In Situ Irradiation NMR Spectroscopy. Chemistry – A European Journal.

[25] Mills A., O’Rourke C. (2015). In Situ, Simultaneous
Irradiation and Monitoring of
a Photocatalyzed Organic Oxidation Reaction in a TiO2-Coated NMR Tube. J. Org. Chem..

[26] Wolff C., Kind J., Schenderlein H., Bartling H., Feldmeier C. (2016). Studies of a photochromic model system using NMR with ex-situ and
in-situ irradiation devices. Magn. Reson. Chem..

[27] Dolinski N. D., Page Z. A., Eisenreich F., Niu J., Hecht S., Read de Alaniz J., Hawker C. J. (2017). A Versatile Approach
for In Situ
Monitoring of Photoswitches and Photopolymerizations. ChemPhotoChem..

[28] Wang H., Li Q., Dai J., Du F., Zheng H., Bai R. (2013). Real-Time
and in Situ Investigation of “Living”/Controlled Photopolymerization
in the Presence of a Trithiocarbonate. Macromolecules.

[29] Liu Y., Xu W. Z., Charpentier P. A. (2020). Reactivity
Ratios of MMA and N,N-Dimethyl-N-{2-[(2-methylprop-2-enoyl)­oxy]­ethyl}­undecane-1-aminium
Bromide in Thermal and UV Initiation Copolymerization. Ind. Eng. Chem. Res..

[30] Hooper T. J. N., de Oliveira-Silva R., Sakellariou D. (2025). High-resolution
in situ photo-irradiation MAS NMR: application to the UV-polymerization
of n-butyl acrylate. Journal of Materials Chemistry
A.

[31] Zhang Z., Zheng H., Huang F., Li X., He S. (2016). Template Polymerization of a Novel Cationic Polyacrylamide: Sequence
Distribution, Characterization, and Flocculation Performance. Ind. Eng. Chem. Res..

[32] El-Newehy M. H., Al-Deyab S. S., Al-Hazmi A. M. A. (2010). Reactivity ratios
for organotin copolymer
systems. Molecules.

[33] Cankaya N., Mürsit Temüz M. (2014). Monomer Reactivity
Ratios Of Cellulose
Grafted With N-Cyclohexylacrylamide And Methyl Methacrylate By Atom
Transfer Radical Polymerization. Cellulose Chemistry
and Technology.

[34] Anastasio R., Maassen E. E. L., Cardinaels R., Peters G. W. M., van
Breemen L. C. A. (2018). Thin film mechanical characterization of UV-curing
acrylate systems. Polymer.

[35] Bagheri A., Jin J. (2019). Photopolymerization
in 3D Printing. ACS Applied
Polymer Materials.

[36] Nakamura A., Munakata K., Ito S., Kochi T., Chung L. W. (2011). Pd-catalyzed copolymerization of methyl acrylate with carbon monoxide:
Structures, properties and mechanistic aspects toward ligand design. J. Am. Chem. Soc..

[37] Litvinov V. M., Dias A. A. (2001). Analysis of network structure of UV-cured acrylates
by 1H NMR relaxation, 13C NMR spectroscopy, and dynamic mechanical
experiments. Macromolecules.

[38] Odian, G. Principles of Polymerization; Wiley-Interscience, 2004. 10.1002/047147875X.

[39] Luu T. T. H., Jia Z., Kanaev A., Museur L. (2020). Effect of Light Intensity
on the Free-Radical Photopolymerization Kinetics of 2-Hydroxyethyl
Methacrylate: Experiments and Simulations. J.
Phys. Chem. B.

[40] Worzakowska M. (2023). Experimental
studies on the preparation and properties of starch-graft-poly­(hexyl
acrylate) copolymers. J. Appl. Polym. Sci..

[41] Schnell M., Borrajo J., Williams R. J. J., Wolf B. A. (2004). Isobornyl methacrylate
as a reactive solvent of polyethylene. Macromol.
Mater. Eng..

[42] Sekine Y., Ikeda-Fukazawa T. (2010). Temperature dependence of the structure of bound water
in dried glassy poly-N,N-dimethylacrylamide. J. Phys. Chem. B.

