# Supplementary material for: In Situ NMR to Monitor Bulk Photopolymerization Kinetics
Source: ACS Macro Lett. 2025 Jun 4;14(6):847–52. doi: 10.1021/acsmacrolett.5c00171 (PMC12177938; doi:10.1021/acsmacrolett.5c00171)
Supplement: Supplementary file 1 [file mz5c00171_si_001.pdf]

In Situ NMR to Monitor Bulk Photopolymerization Kinetics – SUPPORTING INFORMATION

*Luis L. Jessen†, Kameron R. Hansen†, George B. Crull‡, Tanner L. Grover†, C. Allan Guymon**

†Department of Chemical and Biochemical Engineering, The University of Iowa, Iowa City, Iowa, 52242, U.S.A

‡ Department of Chemistry, The University of Iowa, Iowa City, Iowa, 52242, U.S.A

**Department of Chemical Engineering, Brigham Young University, Provo, Utah, 84602, U.S.A*

**Corresponding Author: allan.guymon@byu.edu*

Experimental

Materials

Reagents for in-situ photopolymerizations in the outer NMR tube included monomers hexyl acrylate (HA, Sigma), N,N-dimethylacrylamide (AAm, Sigma), isobornyl methacrylate (IBOMA, Sigma) polyethylene glycol diacrylate (PEGDA, Sigma), and photo initiator diphenyl(2,4,6-trimethylbenzoyl)phosphine oxide (TPO, Darcour). The inside of the concentric capillary was filled with acetic acid (AA, Sigma) dissolved in D_2O (Sigma). Formulations were made by dissolving 1 wt% TPO in monomer and protected from ambient light using aluminum foil to avoid unintentional polymerizations. All chemicals were used as received.

Methods

In-situ apparatus

85 µL of monomer formulation was pipetted into an NMR tube (OD: 5 mm; ID: 4.2 mm). A concentric capillary (New Era NE-5-CIC; OD = 4 mm; ID = 3.5 mm) containing acetic acid (0.24 M) in D_2O (125 µL) was placed inside the NMR tube resulting in a thin layer (~ 0.1 mm) of photocurable resin between the capillary and the outer tube. D_2O was used due to its high boiling point and deuterium density, as well as its minimal absorption in the

wavelength regions used to initiate the reaction (395 nm). To direct light to the sample, a 395 nm LED light (Thorlabs M395F3) powered by a Thorlabs upLED driver was coupled to a fiber optic cable (Thorlabs FP1000URT; 6 m length) using an SMA connection, and the fiber tip was inserted into the concentric capillary inside the spectrometer. The fiber optic was centered within the capillary using a small loop of electrical tape as a spacer. A 15 mm segment of the fiber tip was roughened with 120 grit sandpaper while the uniformity of the output was monitored qualitatively by visual inspection and quantitatively with a light intensity meter at the outer surface of the concentric inner capillary (sample surface). The intensity of light exiting the tip was controlled by adjusting the amperage at the LED driver and measured by averaging the reading over eight locations along the frosted tip that lie within the volume measured by the NMR probe (Figure S2).

In-situ photocure

For all polymerizations, monomer conversion was calculated by monitoring the depletion of vinylic proton resonances at about 6.1 ppm (^{1}H-spectrum, acrylate) or 129.2 ppm (^{13}C-spectrum, acrylate) throughout the polymerization. In between timed bursts of light exposure, spectra were recorded. Using equation 1, conversion of functional groups was then graphed as a function of exposure time:

$$X = 100 * \left(1 - \frac{A_t}{A_i}\right) \tag{1}$$

Here A_t and A_i are the areas of the vinylic proton resonance at time t and 0, respectively. For reference, the ^{1}H-peaks were normalized to the H_2O resonance at 4.7 ppm and ^{13}C-peaks were normalized to the acetic acid resonance at 20.6 ppm. Due to slight increases in sample temperature following the exothermic reaction during light exposure, samples were allowed 30 seconds to return to the target temperature (300 K) before spectra were collected.

NMR Parameters

All experiments were performed on a Bruker Avance Neo 500 (500.3 MHz). The spectrometer was controlled, and the data processed using Topspin 4.3 (Bruker Biospin). A 5 mm BBO probe was used for data collection. The temperature was maintained at 300 K (nominal) using a heated air stream. T1 values for ^{1}H and ^{13}C resonances were obtained using a saturation recovery sequence. The ^{1}H data was acquired with 8 scans with a 30° excitation pulse and a total recycle time of 16 seconds (>5 times T1 of the vinylic ^{1}H resonance used for quantification). 64K complex data points were acquired, and zero filled to 128K before processing. The spectral width was 11 ppm

centered at 4 ppm. An exponential weighting of 0.3 Hz was applied prior to transforming.

The ^{13}C spectra were acquired with 8 scans using an inverse proton decoupled pulse sequence to suppress nOe buildup with a 90° excitation pulse and a total recycle time of 61 seconds (>5 times the T1 of the vinylic ^{13}C resonance used for quantification). 64K complex data points were acquired, and zero filled to 128K before processing. The spectral width was 250 ppm centered at 120 ppm. An exponential weighting of 1 Hz was applied prion to transforming. 8 scans were coadded before transforming. The experimental time was 16 minutes.

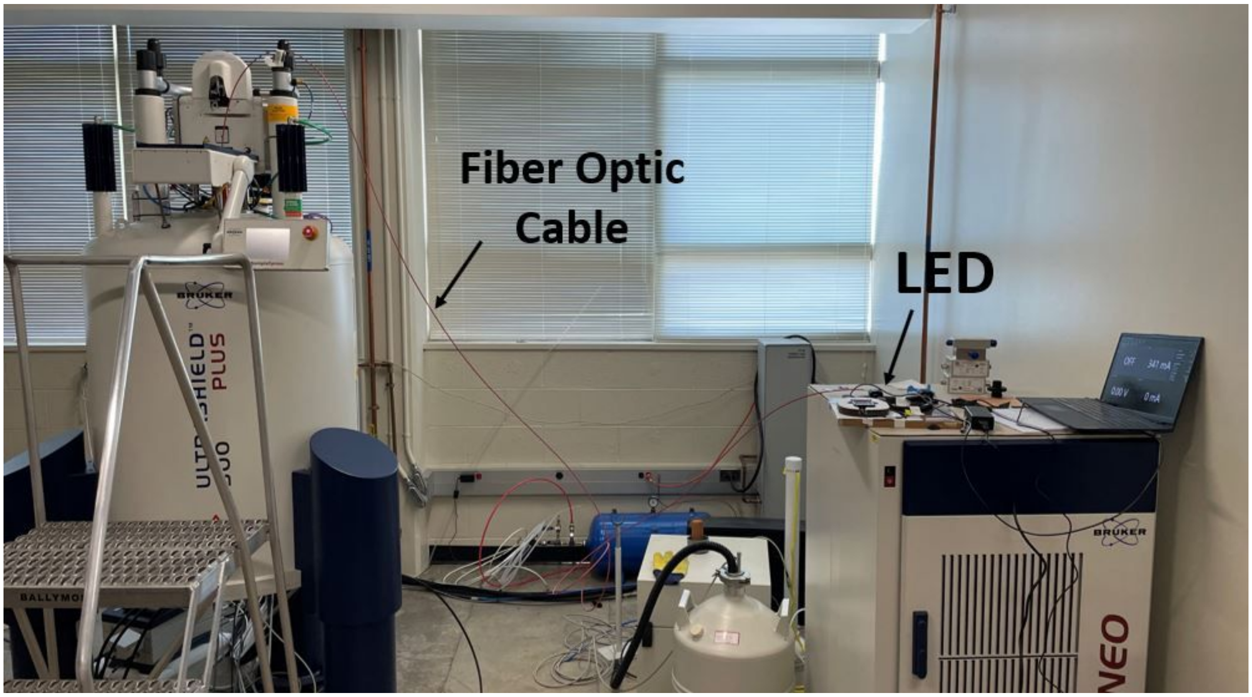

Figure S1. In-situ NMR photopolymerization setup.

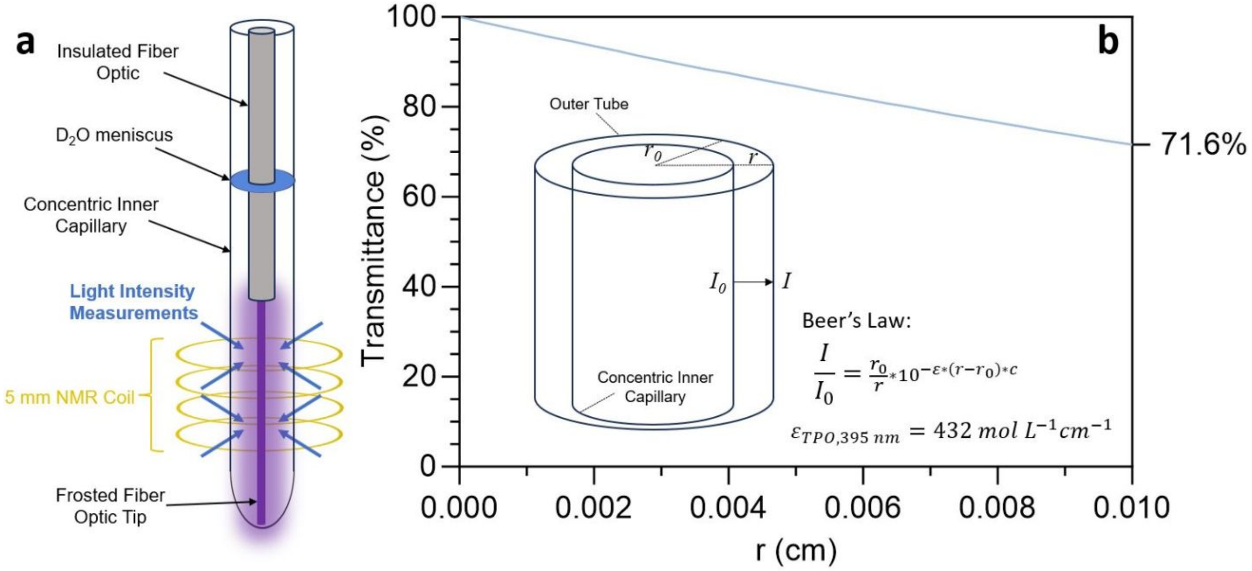

Figure S2. a) Graphic depicting locations of light intensity measurements (blue arrows) along the surface of the concentric inner capillary that is in direct contact with the resin during photocure. Typical relative standard deviations over all 8 locations were typically around 15%. b) Beer's law %-transmittance profile through the thickness of the annulus of a 4-mm concentric inner capillary inside a 5-mm NMR tube (0.1 mm annulus).[1]

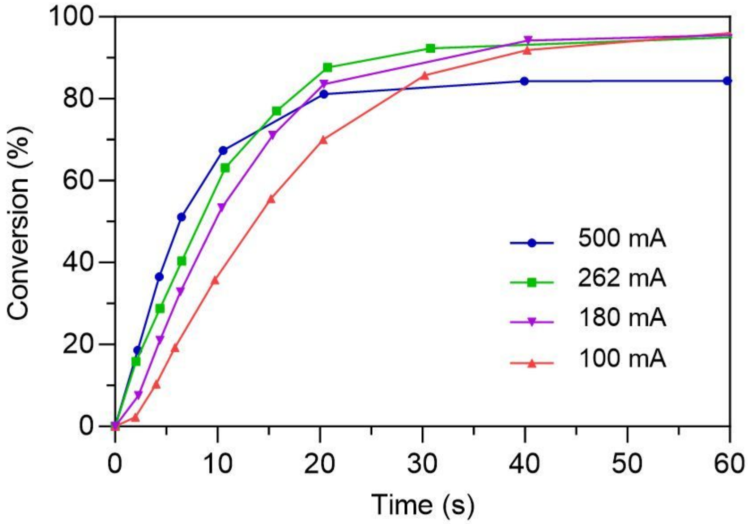

Figure S3. Conversion profiles of hexyl acrylate systems cured at various light intensities.

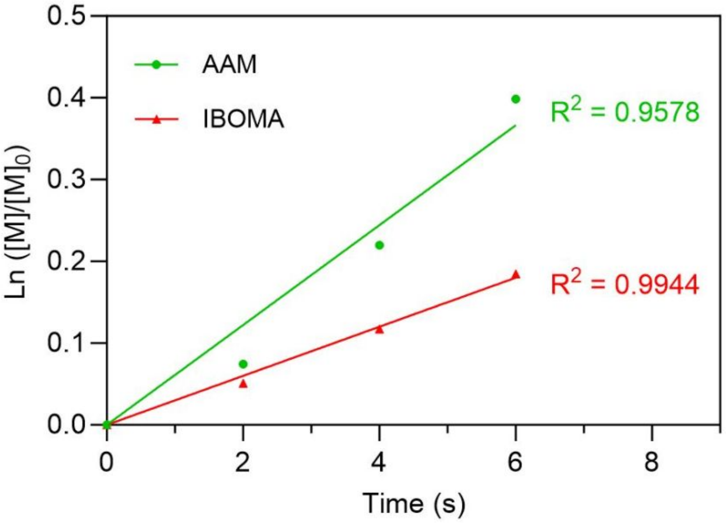

Figure S4. Linearized conversion profiles show an approximately linear relationship predicted by steady state assumption.

References

Sinko, J. E.; Oh, B. I. The Bouguer-Lambert-Beer Absorption Law and Non-Planar Geometries. *AIP Conference Proceedings* **2011**, *1402* (1), 245-257. DOI: 10.1063/1.3657031 (acccessed 5/13/2025).
